# OptDesign: Identifying Optimum Design Strategies in Strain Engineering for Biochemical Production

**DOI:** 10.1021/acssynbio.1c00610

**Published:** 2022-04-07

**Authors:** Shouyong Jiang, Irene Otero-Muras, Julio R. Banga, Yong Wang, Marcus Kaiser, Natalio Krasnogor

**Affiliations:** † Department of Computing Science, University of Aberdeen, Aberdeen AB24 3FX, U.K.; ‡ Institute for Integrative Systems Biology, UV-CSIC, Valencia 46980, Spain; § Computational Biology Lab, MBG-CSIC, Pontevedra 36143, Spain; ∥ School of Automation, Central South University, Changsha 410083, China; ⊥ School of Medicine, University of Nottingham, Nottingham NG7 2RD, U.K.; # School of Computing, Newcastle University, Tyne NE4 5TG, U.K.

**Keywords:** growth-coupled design, flux change, genome-scale metabolic model, systems biology, in silico strain design, biotechnology

## Abstract

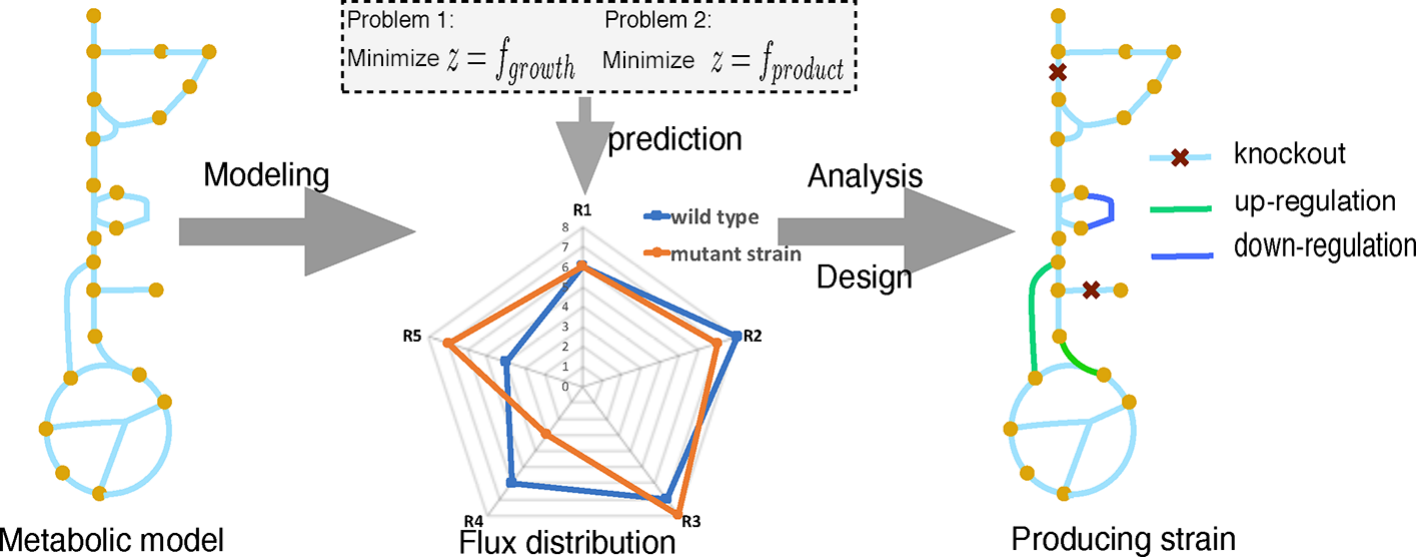

Computational tools have been widely adopted for strain optimization in metabolic engineering, contributing to numerous success stories of producing industrially relevant biochemicals. However, most of these tools focus on single metabolic intervention strategies (either gene/reaction knockout or amplification alone) and rely on hypothetical optimality principles (e.g., maximization of growth) and precise gene expression (e.g., fold changes) for phenotype prediction. This paper introduces OptDesign, a new two-step strain design strategy. In the first step, OptDesign selects regulation candidates that have a noticeable flux difference between the wild type and production strains. In the second step, it computes optimal design strategies with limited manipulations (combining regulation and knockout), leading to high biochemical production. The usefulness and capabilities of OptDesign are demonstrated for the production of three biochemicals in *Escherichia coli* using the latest genome-scale metabolic model iML1515, showing highly consistent results with previous studies while suggesting new manipulations to boost strain performance. The source code is available at https://github.com/chang88ye/OptDesign.

## Introduction

A growing population and fast economical development are leading to an increasing demand of various daily products and industrial raw materials, many of which are derivatives of oil and petroleum. Over the past decades, important efforts are being made to develop sustainable production processes that convert biomass or other renewable resources to bioproducts through cell platforms.^1^ A key challenge in this respect is the design of high-performance strains with efficient metabolic conversion routes to desired products. Recent advances in genome-scale metabolic modeling (GSMM)^2^ have made it possible to have a system-level understanding of cell physiology and metabolism, leading to rational prediction of metabolic interventions for strain development. Systems strain design^1^ has helped to improve the production of numerous biochemicals, including lycopene,^3^ malonyl-CoA,^4^ alkane and alcohol,^5^ and hyaluronic acid.^6^

A number of tools have been developed for strain design.^7,8^ OptKnock,^9^ which was developed to block some reactions in metabolic networks, is one of the earliest such tools. OptKnock identifies the knockout targets that lead to maximal biochemical production in the context of flux balance analysis^2^ that is subject to mass balance and thermodynamic constraints. This results in a bilevel optimization problem which can be solved through mathematical reformulation into a standard mixed-integer linear program (MILP).^9^ The OptKnock model was later extended to consider gene up/down-regulation,^10^ swap of cofactor specificity,^11^ and introduction of heterologous pathways^12^ for biochemical production. It was also adapted to identify synthetic lethal genes for anti-cancer drug development.^13^ Some improvement strategies, such as GDBB^14^ and GDLS,^15^ have been proposed to improve the efficiency of OptKnock in solving the bilevel problem. There also exist numerous approximate solutions to the OptKnock model, including genetic algorithms^16^ and swarm intelligence.^17^ Designing strains that couple production to growth has received increasing attention in recent years, mainly due to the great production potential of growth-coupled strains in adaptive laboratory evolution.^18^ Consequently, a number of computational tools along this direction have been developed to design strains with various growth-coupled phenotypes.^19,20^ OptCouple^20^ simulates jointly gene knockouts, insertions, and medium modifications to identify growth-coupled designs, although gene expression regulation is not considered. In addition, game theory has been introduced into metabolic engineering.^21,22^ NIHBA^22^ considers metabolic engineering design as a network interdiction problem involving two competing players (host strain and metabolic engineer) in a max-min game enabling growth-coupled production phenotypes, and the problem is solved by an efficient mixed-integer solver. Furthermore, there are also some studies which do not rely on optimality principles for phenotype prediction. Among these, the minimum cut set (MCS)-based approach,^23,24^ which aims to find the smallest number of interventions blocking undesired production phenotypes, has been extensively studied. Despite high computational complexity, MCS-based approaches have successfully predicted strain design strategies leading to in vivo biochemical production.^25^ Another important approach of the same kind is OptForce, which identifies metabolic interventions by exploring the difference in flux distributions between the wild type and the desired production strain.^26^ OptForce has shown good predictions for in vivo malonyl-CoA production.^4^

The use of computational tools is of undisputed importance to strain development in metabolic engineering.^27^ However, there are several limitations which may prevent the wide applicability of the above-mentioned approaches. First, most of the tools focus on prediction of either knockout targets or regulation targets alone, with a few exceptions that are capable of predicting both interventions, such as OptForce^26^ and OptRAM.^28^ These exceptions highlight that a combination of knockout and up/down-regulation often leads to higher biochemical production compared to a single strategy. OptForce encourages the use of flux measurements while identifying optimum design strategies. OptRAM considers regulatory networks from which transcriptional factors can be optimized for biochemical production. However, both OptForce and OptRAM rely heavily on the precise expression level of regulation targets; for example, desired production phenotypes can only be achieved at the exactly suggested flux values (OptForce) or up/down-regulation fold changes (OptRAM). It is known that gene expression is a complex process with many uncertainties. The underlying strict expression requirements in these approaches may miss theoretically non-optimal but practically feasible design strategies. In addition, both approaches rely on a reference flux vector of the wild type, which can be incorrectly chosen from many steady-state flux distributions if it cannot be uniquely determined. Second, many existing strategies rely on the assumption of optimality principles, for example, maximal growth in OptKnock^9^ and derivatives, in the cell metabolism. However, this assumption is not always an accurate representation of how cells respond to metabolic perturbations or environmental changes.^29^ NIHBA^22^ showed that reducing unnecessary surrogate biological objectives helps to identify many non-optimal but biologically meaningful knockout solutions.

This paper introduces a new computational tool, called OptDesign, that uses a two-step strategy to predict rational strain design strategies for biochemical production. OptDesign has the following capabilities:

(C1) overcomes the uncertainty problem as there is no assumption of exact fluxes or fold changes that cells should have for production. As a result, non-optimal but good feasible solutions are not missed.

(C2) allows two types of interventions (knockout and up/down-regulation).

(C3) disregards the assumption of (potentially unrealistic) optimal growth in the production mode.

(C4) can use with or without reference flux vectors.

(C5) guarantees growth-coupled production (if desired up/down-regulations are achievable in vivo).

OptDesign is the only tool that combines these five capabilities, as shown in Table 1. In the remainder of this paper, we describe OptDesign and benchmark it considering three case studies, demonstrating high consistencies of predicted design strategies with previous in vivo and in silico studies.

**Table 1:** Comparison of Different Strain Design Tools^a^

tool	C1	C2	C3	C4	C5
OptKnock^b^	×	×	×	√	×
OptForce^26^	×	√	×	×	×
OptCouple^20^	×	×	×	√	√
OptReg^10^	×	√	×	√	×
OptRAM^28^	×	√	×	×	×
NIHBA^22^	√	×	√	√	√
OptDesign (this study)	√	√	√	√	√

a (C1) overcomes the uncertainty problem as there is no assumption of exact fluxes or fold changes that cells should have for production, (C2) allows two types of interventions (knockout and up/down regulation), (C3) disregards the assumption of optimal growth in the production mode, (C4) can use with or without reference flux vectors, and (C5) guarantees growth-coupled production (if desired up/down-regulations are achievable in vivo).

b The original OptKnock may not always achieve growth-coupled production, but its derivative RobustKnock^30^ is guaranteed to achieve this.

## Materials and Methods

A metabolic network of *m* metabolites and *n* reactions has a stoichiometric matrix *S* that is formed by stoichiometric coefficients of the reactions. Let *J* be a set of *n* reactions and *v*_*j*_ the reaction rate of *j* ∈ *J*; *Sv* represents the concentration change rates of the *m* metabolites. The flux space FS is defined as the space spanned by all possible flux distributions *v* for the system subject to thermodynamic constraints at steady state (i.e., the concentration change rate is 0 for all the metabolites). Mathematically, FS can be described as
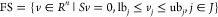
1where lb_*j*_ and ub_*j*_ are the lower and upper flux bounds of reaction *j*, respectively. We use the notation FS_w_ for the wild type and FS_m_ for the mutant strain. Flux balance analysis (FBA) determines a single solution in FS when a surrogate biological objective is provided for 1.

OptDesign recognizes metabolic changes from the wild type to production (mutant) strains. Let *v* ∈ FS_w_ denote a flux vector of the wild type and Δ*v* denotes the flux change needed for *v* to transition into a desired production state. Obviously, *v* + Δ*v* represents a flux vector of the production strain, and it needs to satisfy mass balance and some production requirements, that is, *v* + Δ*v* ∈ FS_m_. Note that flux measurements can be used to customize the flux bounds in FS_w_ and FS_m_ if available; otherwise, the flux bounds can be set according to flux variability analysis (FVA) predictions. For example, FS_m_ can be constrained by imposing production requirements on the lower bounds of the production reaction and biomass.

OptDesign introduces the concept of noticeable flux difference δ (mmol/g_DW_/h) between the wild type strain and the production strain in reactions. OptDesign uses this concept to identify an optimal set of manipulations leading to the production phenotype FS_m_. To do so, OptDesign performs two key steps of optimization. First, OptDesign identifies a minimal set of reactions that must deviate from their wild-type flux with at least δ in order to achieve FS_m_. This set of reactions form candidate regulation targets. Second, OptDesign searches through regulation candidates, together with knockout candidates, for the optimal combination of manipulations to maximize biochemical production. The following two subsections are devoted to presenting these two steps in detail.

### Selecting Up/Down-Regulation Reaction Candidates

**Figure 1:**
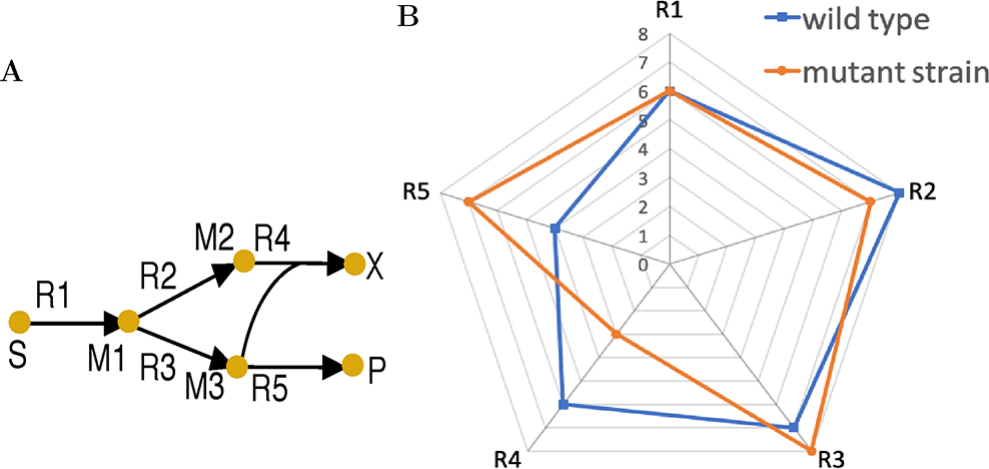
Toy metabolic network (A) and flux distributions of the wild type and mutant (B). Symbols in (A) are as follows: S, carbon source; X, biomass; P, product; M_*i*_ (*i* = 1, 2, 3), metabolite name; R_*i*_ (*i* = 1,..., 5), reaction name. Each axis in (B) represents the absolute flux for a reaction.

This step of OptDesign is to identify the minimum number of reactions whose flux must have a noticeable change if the cellular metabolism shifts from the wild type to the required production state. A reaction is considered a candidate for up-regulation if its flux in the mutant is at least δ units more than that in the wild type. On the contrary, this reaction is considered for down-regulation if its flux in the mutant is at least δ units fewer than that in the wild type. Note that the above directional up/down-regulation definition is used for computational convenience, and final regulation targets identified from OptDesign will be rationally grouped by contrasting the wild type to the mutant strain by their absolute flux values (which will be detailed later in the section Materials and Methods). In any other situations, this reaction is not considered as a candidate for genetic manipulation. Figure 1 illustrates the above concept with a toy network of five reactions. Suppose δ is set to 2 units for all these five reactions, R4 and R5 are considered for down-regulation and up-regulation, respectively. However, R1, R2, and R3 are not selected as regulation candidates since their flux changes from the wild type to the mutant are within the predefined threshold δ.

An MILP procedure is employed to minimize the number of reactions that must change their flux from the wild type to the mutant by at least δ units. This can be expressed as the following MILP problem
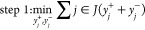
2a
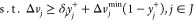
2b
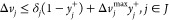
2c
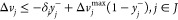
2d
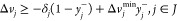
2e
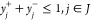
2f
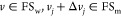
2gwhere *y*_*j*_^+^ and *y*_*j*_^–^ are binary variables representing that the flux of reaction *j* increases and decreases by at least a noticeable level δ_*j*_ > 0 from the wild type to the production phenotype, respectively. Equivalently, *y*_*j*_^+^ = 1 (*y*_*j*_^–^ = 1) implies Δ*v*_*j*_ ≥ δ_*j*_ (Δ*v*_*j*_ ≤ −δ_*j*_). Constraints 2b–2e are for flux increase and decrease, respectively. Δ*v*_*j*_^min^ and Δ*v*_*j*_^max^ are the lower and upper bounds of flux change Δ*v*_*j*_, respectively. Special reactions in which fluxes are not allowed to be decreased (increased), for example, non-growth-associated maintenance, should have a zero value for the lower (upper) bound of their corresponding Δ*v* components. A reaction cannot increase and decrease flux simultaneously, which implies constraint 2f. Constraints 2g describe the flux space of the wild type and mutant strain at steady state, where FS_w_ and FS_m_ are constrained differently by specifying a minimum growth rate and a minimum target production rate, respectively. It is worth noting that the minimum target production is not a requirement for the producing strain. Instead, it is mainly used to identify the set of reactions that must change their flux in order to produce the target compound. In practice, we can fulfil this purpose by setting the minimum target production rate to the maximum theoretical production rate (computed by FBA with an objective to maximize the flux through the reaction acting on the target product).

Note that this procedure does not need a known reference flux vector for both the wild type and the mutant; instead, it takes into account all possible wild-type and mutant flux distributions that meet engineering requirements (e.g., growth rate, production/yield). However, it is recommended to make use of flux measurements for the wild-type and mutant strains if possible in order to select a rational set of regulation targets effectively.

### Identifying Optimal Manipulation Strategies

The solution to the MILP (2) results in a flux-increase set *F*^+^ (corresponding to reactions with *y*_*j*_^+^ = 1) and a flux-decrease set *F*^–^ (corresponding to reactions with .*y*_*j*_^–^ = 1) in addition to the suggested flux change Δ*v*. However, these two sets are not the minimum number of manipulations needed for the required production state as the effects of some manipulations can be propagated to the whole metabolic network.^26^ In addition, there is an engineering cost in manipulating reactions (through gene–protein–reaction associations), and therefore, it is assumed there is a limit on the number *K*_m_ of genetic manipulations, including up/down-regulation and knockout. We allow gene/reaction knockout in this step for two reasons. First, reactions having an unnoticeable flux change (below δ_*j*_ and thus not in *F*^+^ or *F*^–^) may sometimes be good manipulation targets, especially when they are involved in completing pathways. Taking them as potential knockout targets could improve biochemical production (essentially a relaxation of optimization models). Second, there may be reactions in the regulation candidate set that carry near-zero fluxes in the production strain. From a practical point of view, completely deactivating them by gene knockout is easier than regulating their gene expression precisely to the suggested minute fluxes. Reaction knockout candidates (denoted by set *F*^×^) can be selected by a preprocessing approach,^22^ which excludes reactions that are essential, irrelevant, or unlikely to be good knockout targets.

Here, we treat the strain design task as a network interdiction problem^22^ that maximally forces cells to violate their wild-type phenotypes for production, that is, to choose the optimum manipulations from *F*^+^ ∪ *F*^–^ ∪ *F*^×^ in favor of biochemical production regardless of what the wild-type flux distribution is. As a result, we develop the following network interdiction problem
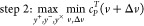
3a
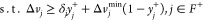
3b
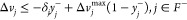
3c
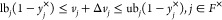
3d
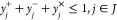
3e
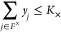
3f
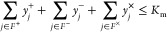
3g
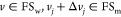
3hwhere *c*_P_ is a coefficient vector for the target biochemical. *y*_*j*_^×^ = 1 represents the knockout of reaction *j*, leading to zero flux in this reaction as illustrated by constraint 3b. Constraints 3f and 3g limit the allowable number of knockouts and the total number of manipulations.

The above statement is a special bilevel problem and can be formulated to a standard MILP using duality theory^9^ (see reformulation in Supporting Information Data 1). The resulting MILP can be handled either by a modern MILP solver or, if numerous alternative solutions are desired in a single run, by the hybrid Benders algorithm.^22^

The solution to problem 3 contains some regulation-associated binary variables that have a value of 1, that is, *y*_*j*_ = 1, for some *j* ∈ *F*^+^ ∪ *F*^–^. The integer values represent the reaction targets whose flux needs a change for biochemical production. In order to determine how they are to be regulated in experimental implementation, the following classification rule is applied
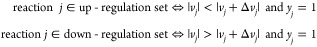


The output of the model (3) predicts which reaction should be up- or down-regulated by at least the chosen flux change threshold. It does not impose exact fluxes on the mutant strain to guarantee the high production of target chemicals. In this sense, the resulting manipulations suggested by OptDesign could be experimentally more feasible than those obtained by existing tools.

### Computational Implementation

OptDesign relies on model reduction and candidate selection for computational efficiency. Genome-scale metabolic (GEM) models can be significantly simplified by compressing linearly linked reactions and removing dead-end reactions (those carrying zero fluxes). Similarly, many reactions can be excluded from consideration with a priori knowledge that, for example, they are vital for cell growth or their knockout is not likely to improve target production. We followed the model reduction and candidate selection procedure^22,31^ (the detailed procedure can be found in Supporting Information Data 1), resulting in a much smaller knockout candidate set for each target product in the latest *Escherichia coli* GEM iML1515.^32^ The flux change threshold δ_*j*_ = 1 mmol/g_DW_/h was used throughout the paper unless otherwise stated. Algorithm 1 presents the pseudocode of OptDesign. It was implemented in MATLAB 2018b to be compatible with the Cobra Toolbox 3.0.^33^ All MILPs were solved by Gurobi 9.02.^34^ It is worth noting that a couple of minutes is enough for a modern optimization software like Gurobi to identify a reasonably small set of up/down-regulation candidates. The source code is available for download at https://github.com/chang88ye/OptDesign.
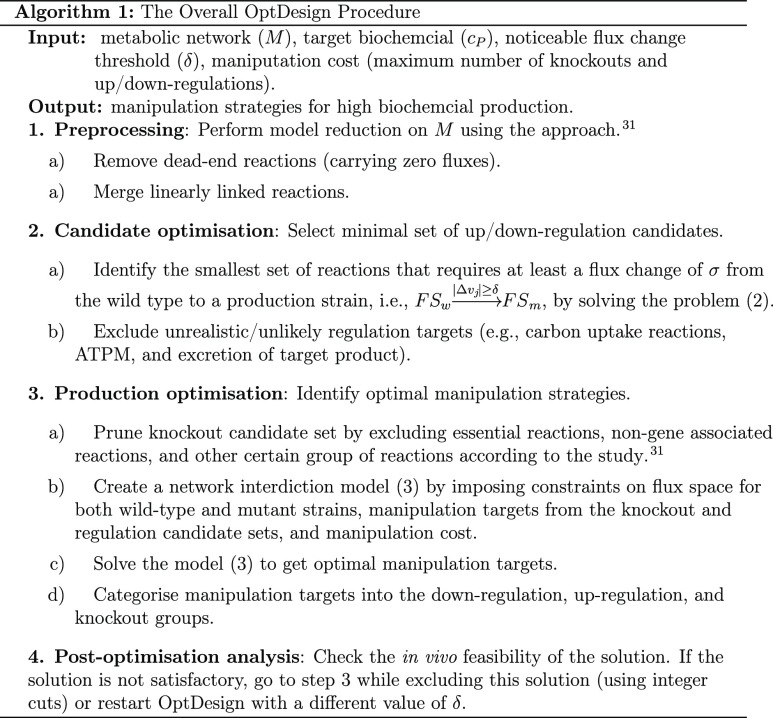


## Case Studies

The OptDesign framework was tested by identifying metabolic manipulations for the production of three industrially relevant biochemicals using the latest genome-scale metabolic model iML1515^32^ for *E. coli*. Glucose was used as the sole carbon substrate, and its maximum uptake rate was set to 10 mmol/g_DW_/h. These biochemicals include a number of compounds that have been experimentally studied in the literature. In particular, we focussed on the native succinate and non-native lycopene and naringenin^26,35^ in our case studies. A comparison between OptDesign predictions and experimentally validated interventions for another nine target biochemicals can be found in Supporting Information Data 2. The heterologous biosynthesis pathway added to iML1515 for the production of two non-native biochemicals can be found in Supporting Information Data 1. The newly added reactions were charge- and mass-balanced. The growth conditions were the same as in iML1515, except that a minimal cell growth of 0.1 h^–1^ was imposed on mutant strains for biochemical production.^31^ All the other parameters remained the same as the original iML1515.^32^ At most 10 manipulations including no more than 5 knockouts were allowed, and the restriction on knockout is to intentionally favor gene expression manipulation over gene knockout. All optimization problems in OptDesign were solved by Gurobi 9.02 on a MacBook with a 3.3 GHz Intel Core i5 processor and 16 GB RAM. The optimization process was terminated by multiple stopping criteria whichever was met first, including the time limit (10^4^ seconds) and optimality gap (5%). Indeed, we observed that the incumbent solution did not improve either after 3000 s or when the optimality gap reached 5%.

### Case Study 1: Succinate Overproduction

**Figure 2:**
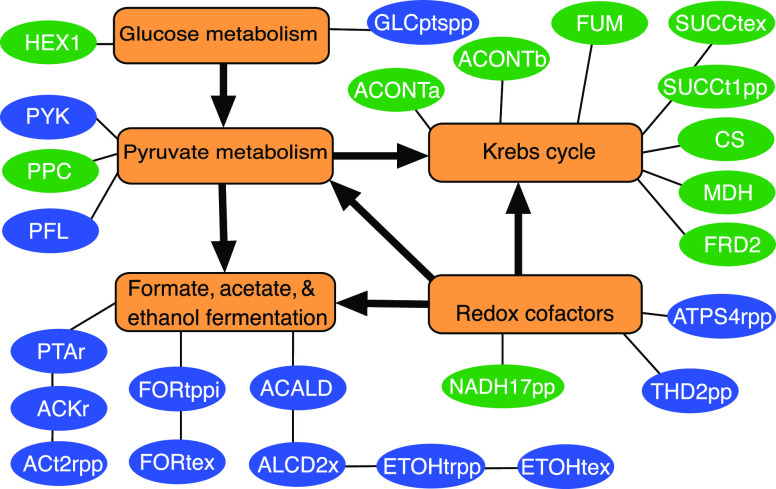
Reactions identified as up/down-regulation targets by OptDesign for succinate overproduction. Abbreviations of reaction names are borrowed from the iML1515 model definitions. Up-regulation and down-regulation reactions are in green and blue ovals, respectively. These reactions have been classified into different subsystems represented by orange rectangles.

As a starting point, we wondered which reactions are likely to be good regulation targets and how they are distributed in metabolic networks. Therefore, we extracted the candidate regulation targets from the first step of OptDesign and reorganized them into different metabolic subsystems, as shown in Figure 2. It can be observed that the majority of regulation candidates are from the Krebs cycle and fermentation products (i.e., formate, acetate, and ethanol) that have the same precursor acetyl-CoA as succinate. It is suggested that all the reaction candidates from the Krebs cycle should increase their flux and those related to the formation of formate, acetate, and ethanol should lower their activity. This prediction is consistent with many studies of succinate production.^26,36,37^ In addition to these two main subsystems, reactions from the glucose metabolism, pyruvate metabolism, and cofactor conversion/formation are also possible regulation targets. For example, the glucose transporter (GLCptspp) predicted for down-regulation here has been a deletion target in another study^38^ to enhance succinate production.

**Figure 3:**
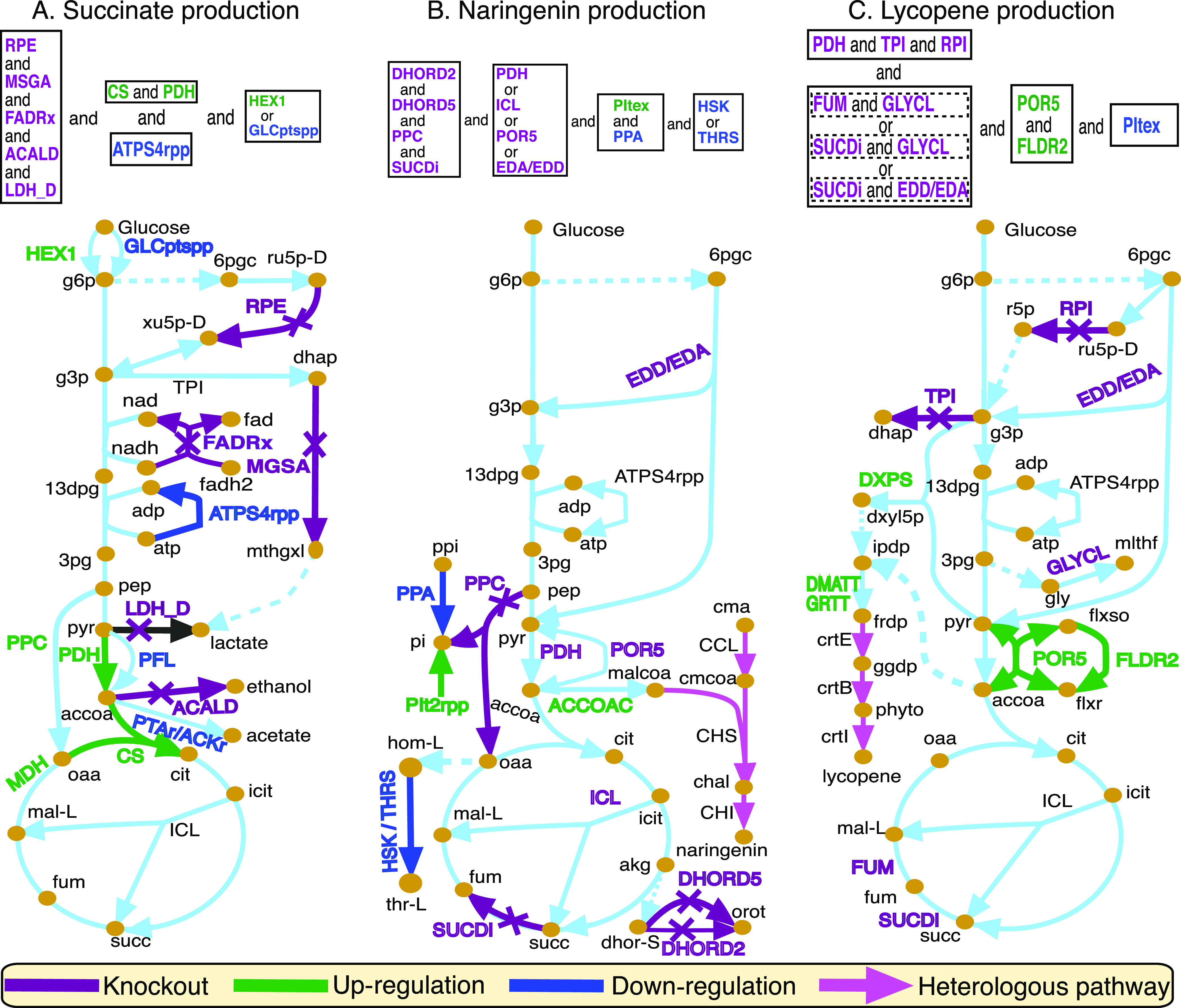
Design strategies identified by OptDesign for biochemical production in *E. coli*. Reaction names and their arrow symbols in the same color mean that they must be manipulated in mutant strains. Reaction names colored only (i.e., red, green, or blue) mean that they are alternative manipulations. Dashed arrows represent a merge of multiple conversion steps to metabolites. Design strategies are summarized in boxes above the simplified metabolic maps. Abbreviations of metabolite names are as follows: g6p, glucose-6-phosphate; f6p, d-fructose 6-phosphate; g3p, glyceraldehyde-3-phosphate; 13dpg, 3-phospho-D-glyceroyl phosphate; 3gp, 3-phospho-d-glycerate; 6pgc, 6-phospho-d-gluconate; ru5p-D, d-ribulose 5-phosphate; r5p, alpha-d-ribose 5-phosphate; xu5p-D, d-xylulose 5-phosphate; dhap, dihydroxyacetone phosphate; mthgxl, methylglyoxal; pep, phosphoenolpyruvate; pyr, pyruvate; lac-D: d-lactate; dxyl5p, 1-deoxy-d-xylulose 5-phosphate; ipdp, isopentenyl diphosphate; frdp, farnesyl diphosphate; ggdp, geranylgeranyl diphosphate; phyto, all-trans-phytoene; ppi, diphosphate; pi, phosphate; gly, glycine; mlthf, 5,10-methylenetetrahydrofolate; flxso, flavodoxin semi oxidized; flxr, flavodoxin reduced; accoa, acetyl-CoA; cit, citrate; icit, isocitrate; akg, 2-oxoglutarate; succ, succinate; fum, fumarate; mal-L, l-malate; oaa, oxaloacetate; hom-L, l-homoserine; thr-L, l-threonine; dhor-S, (S)-dihydroorotate; orot, orotate; malcoa, malonyl-CoA; cma, coumaric acid; cmcoa, coumaroyl-CoA; chal, naringenine chalcone; fad, flavin adenine dinucleotide oxidized; fadh2, flavin adenine dinucleotide reduced. Abbreviations of reaction names are referred to the iML1515 model definitions.

Figure 3a shows a few final design strategies identified by OptDesign that can improve succinate production. It suggests eight primary manipulations, including the knockout of five reactions, up-regulation of citrate synthase (CS) and pyruvate dehydrogenase (PDH), and down-regulation of periplasmic ATP synthase (ATPS4rpp). Two crucial enzymes in the formation of the fermentation products lactate and ethanol, that is, lactate dehydrogenase (LDH_D) and acetaldehyde dehydrogenase (ACALD), are suggested to be deactivated as they are considered as competing pathways consuming succinate precursors. These two manipulation targets have been observed in several studies.^39,40^ The knockout of FAD reductase (FADRx) increases the availability of NADH, which has shown to be an effective approach to high succinate production.^41^ The methylglyoxal synthase (MGSA) pathway to lactate is another primary knockout target predicted by OptDesign. The removal of this minor pathway should result in pyruvate accumulation for succinate biosynthesis. Interestingly, this knockout has been implemented in previous studies,^37,40^ resulting in an increased flux in the Krebs cycle. Ribulose-phosphate 3-epimerase (RPE) is another manipulation target identified by OptDesign, whose knockout blocks the conversion of ribose-5-phosphate (R5P) to d-xylulose 5-phosphate (xu5p-D) in the pentose phosphate pathway.^42^ Therefore, it is expected that primary glycolytic flux flows into the precursors, for example, phosphoenolpyruvate and pyruvate, of succinate.

OptDesign suggests to overexpress two enzymes in the succinate biosynthetic pathway, that is, PDH and CS, which are intuitively straightforward to understand. In anaerobic *E. coli*, the PDH activity is either low or undetectable in order to maintain redox balance.^43^ However, it is observed that an *E. coli* mutant with activation of PDH for extra NADH improves succinate production.^40^ Overexpression of CS, which is also suggested in another study,^26^ has been observed to increase the flux in the Krebs cycle in a malic acid production *E. coli* strain.^44^

OptDesign further predicts that high succinate production requires either up-regulation of glucokinase (HEX1) or down-regulation of a phosphoenolpyruvate-dependent phosphotransferase system (PTS)-related reaction (GLCptspp), both of which have the same effect that glucose transport is favorably through the ATP-consuming HEX1 rather than the more efficient but phosphoenolpyruvate-dependent PTS route. Consequently, it improves the availability of PEP, which is a precursor for biomass formation and many biochemicals including succinate. Both manipulation approaches have been observed to improve the succinate yield.^36^ However, the increased ATP demand due to glucose transport via HEX1 has to be mediated by increased ATP production by other means. For this reason, OptDesign suggests down-regulation of ATPS4rpp to reduce cleavage of ATP to ADP in order to meet metabolic energy requirements. This prediction has been also suggested in another study.^45^

OptDesign also identifies a number of additional modification targets, such as pyruvate formate lyase (PFL), phosphotransacetylase (PTAr), and acetate kinase (ACKr), that have been widely used as knockouts to increase the flux toward the Kreb cycle in succinate-focused studies.^37,40^ However, here, OptDesign suggests to down-regulate these enzymes instead of deactivating them completely. In addition, phosphoenolpyruvate carboxylase (PPC) and malate dehydrogenase (MDH) are also predicted as promising overexpression targets. This result is consistent with experimental studies that show increased succinate production through up-regulating these two enzymes.^40,46^

### Case Study 2: Naringenin Production

A three-step pathway for naringenin was introduced into the metabolic network *E. coli* (see Figure 3b), and unlimited coumaric acid (cma) was supplemented in the growth medium.^35^ OptDesign predicts that naringenin production requires four primary knockouts, one up-regulation and two down-regulations. The first two primary knockouts are dihydroorotic acid dehydrogenases (DHORD2 and DHORD5) that catalyze the oxidation of dihydroorotate to orotate in the pyrimidine biosynthesis pathway. The knockout of the underlying gene pyrD for these two reactions results in a reduced growth rate,^47^ which might save the carbon source for naringenin biosynthesis. The knockout of succinate dehydrogenase (SUCDi) creates a surplus of the biosynthetic precursor acetyl-CoA for naringenin, which has been experimentally observed in a previous study.^35^ Phosphoenolpyruvate carboxylase (PPC), a metabolic shortcut for the conversion of phosphoenolpyruvate to oxaloacetate and the byproduct phosphate, is also listed as a primary knockout. We postulate that in addition to avoid the accumulation of phosphate, its deletion could not only direct the flux through pyruvate to acetyl-CoA but also reduce the consumption of acetyl-CoA in the Krebs cycle for the mediation of oxaloacetate. In fact, PPC mutants were found to have a flux increase from pyruvate to acetyl-CoA in a ^13^C-labeling experiment.^48^ Two linear reactions, that is, threonine synthase (THRS) and homoserine kinase (HSK), which are involved in the formation of l-threonine from l-homoserine, are predicted as down-regulation targets. This manipulation is expected to reduce carbon consumption in competing pathways, which therefore increases the carbon flux toward naringenin. Another down-regulation target is the inorganic diphosphatase (PPA) that catalyzes the conversion of one ion of pyrophosphate to two phosphate ions. This manipulation is not intuitively straightforward and believed to create a combined effect with other manipulations to boost naringenin production. Since PPA down-regulation produces less phosphate which is needed in the added naringenin biosynthesis pathway, phosphate has to be balanced through an increase in its transport channel, that is, the phosphate transporter (PItex).

Aside from the above primary manipulations, it is also predicted that naringenin production strains must block at least one of the following reactions: two reactions on the Entner–Doudoroff pathway (EDD/EDA), pyruvate synthase (POR5), isocitrate lyase (ICL), and PDH. Blocking EDD/EDA might increase the use of glucolycosis, producing more ATP which is needed in the heterologous naringenin pathway. The removal of POR5 or PDH forces *E. coli* to use alternative conversion routes from pyruvate to acetyl-CoA without depleting coenzyme A (CoA), another primary precursor for naringenin biosynthesis. The knockout of ICL prevents the malate synthase reaction from consuming acetyl-CoA. In addition, OptDesign also predicts that the up-regulation of acetyl-CoA carboxylase (ACCOAC) helps to increase the production of naringenin, which has been implemented in another study.^35^

### Case Study 3: Lycopene Production

A non-native lycopene biosynthetic pathway consisting of three key reactions were added to the metabolic network of *E. coli* (see Figure 3c). A preliminary execution of OptDesign predicted the need of only one modification, which is the overexpression of the gene encoding dimethylallyltranstransferase (DMATT) or the one encoding geranyltranstransferase (GRTT). While this manipulation intuitively makes sense, gene overexpression only in the upstream biosynthesis pathway of lycopene does not lead to high lycopene production due to a low concentration of precursors, as experimentally illustrated in another study.^49^ Therefore, we run our tool again while disallowing DMATT/GRTT to be valid regulation targets. Consequently, a variety of design strategies were identified, as shown in Figure 3c. Specifically, all the design strategies are combinations of seven manipulations, consisting of five knockouts, two up-regulations, and one down-regulation. However, they differ from each other in only two knockout targets. The three primary knockouts, that is, ribose-5-phosphate isomerase (RPI), triose-phosphate isomerase (TPI), and PDH, are linked to two precursors (i.e., glyceraldehyde-3-phosphate and pyruvate) of lycopene biosynthesis. The knockout of RPI reroutes the carbon flux flowing into the lycopene precursors using more effective metabolic routes (e.g., glycolysis) rather than the non-oxidative pentose phosphate pathway, which is consistent with the study,^3^ in addition to slowing down cell growth due to reduced ribose-5-phosphate formation for RNA and DNA synthesis. Both TPI and PDH knockouts should immediately increase the availability of the lycopene precursors, with the latter for increased lycopene biosynthesis being already confirmed experimentally in another study.^50^ Apart from glyceraldehyde-3-phosphate and pyruvate, acetyl-CoA is also an important precursor to form isopentenyl diphosphate, a building block for lycopene, using a different pathway. Therefore, it is expected that increasing the availability of acetyl-CoA should also improve lycopene. Unsurprisingly, POR5 is predicted as an up-regulation target in compensation for the loss of PDH for acetyl-CoA formation. Also, reducing the amount of acetyl-CoA flowing into the Krebs cycle was found to increase the flux toward isopentenyl diphosphate.^51^ This is fulfilled by removing either fumarase (FUM) or SUCDi in this study. Each of these two knockouts has to be paired with an additional knockout outside the Krebs cycle. This leads to three most frequent pairs, that is, the glycine cleavage system (CLYCL) with FUM, CLYCL with SUCDi, and SUCDi with EDD/EDA. The predicted CLYCL knockout is believed to help reduce the cleavage of 3-phospho-d-glycerate into the glycine biosynthetic pathway so that more pyruvate can be accumulated. Alternatively, blocking the Entner–Doudoroff pathway allows more flux into glycolysis, leading to a higher production of the two precursors (i.e., glyceraldehyde-3-phosphate and pyruvate) for lycopene.

The NADPH-dependent flavodoxin reductase (FLDR2) is another primary up-regulation target predicted by OptDesign. Overexpressing FLDR2 is thought to balance the significantly increased ratio of NADPH to NADP^+^ caused by the last step of the lycopene biosynthetic pathway. Last, it is predicted that reducing the phosphate uptake rate improves lycopene production. This is probably because two out of the three reactions added for lycopene biosynthesis produce diphosphate that can be converted to phosphate, and a flux decrease in this uptake reaction rebalances phosphate in the system.

## Discussion

This paper has presented a new computational tool, called OptDesign, to aid strain development through rational identification of genetic manipulations including reaction knockout and flux up/down-regulation. This tool has been benchmarked via three case studies of different biochemicals, demonstrating its capability of identifying high-quality strain design strategies to improve biochemical production.

OptDesign predicts well in its first computational step a set of candidates that can be potentially used as experimental regulation targets, as shown in the succinate case. In a second computational step, the algorithm further prunes this set to a realistically acceptable size while optimizing biochemical production. Interestingly, many of the predicted manipulations have been experimentally implemented in previous studies. Taking succinate production as an example, 10 out of 14 manipulations (MSGA, ACALD, LDH_D, HEX1, PDH, PFL, PTAr/ACKr, GLCptspp, PPC, and MDH) suggested by OptDesign have been employed in succinate-producing strains.^36,37,40,46^ Specifically, it has been shown that engineered *E. coli* strains KJ060 and KJ073 produce succinate yields of 1.2–1.6 mol/mol glucose after removing completing pathways that lead to the byproducts ethanol, acetate, formate, and lactate.^40^ These strains were developed through added acetate in culture media because the deletion of PFL causes acetate auxotrophy under anaerobic conditions.^37^ However, OptDesign suggests that there is no need to completely deactivate PFL. Instead, down-regulating it avoids acetate auxotrophy while still achieving high succinate production.^a^ Additionally, it is observed that glucose transport favoring glucokinase over pep-dependent PTS yields higher succinate production.^52^ Furthermore, the overexpression of PPC in *E. coli* for increasing succinate yields has been confirmed in a previous study.^46^

OptDesign also suggests a few new modifications, such as the deletion of FADRx and RPE, the up-regulation of CS, and down-regulation of ATPS4rpp, which to our best knowledge have not been experimentally implemented for succinate production. While up-regulation of CS has been shown to increase malic acid production,^44^ it remains unclear whether this manipulation is also useful for succinate production. The suggested flux modifications on FADRx and ATPS4rpp reconfirm the importance of ATP and redox balance in succinate-producing strains.^40^ Deletion of RPE showed low flux in the Krebs cycle,^53^ suggesting that metabolic bottlenecks may exist upstream of the Krebs cycle. The design strategies predicted by OptDesign imply that a synergistic effect of RPE knockout with the other identified flux modifications can lead to a high production of succinate. Similar observations can also be found for the production of two non-native biochemicals, naringenin and lycopene, studied in this paper.

**Figure 4:**
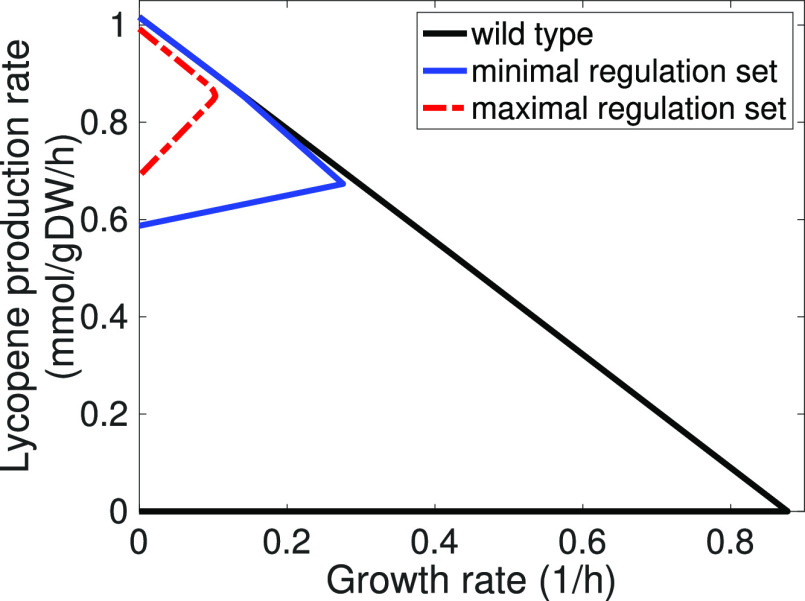
Production envelopes of different growth-coupled design strategies consisting of no more than five manipulations for lycopene. The production envelope illustrates the minimum and maximum production rates a production strain can achieve at different growth rates compared to the wild type. The solid-blue production envelope is for the design strategy using the minimal regulation set: ALCD19 (knockout), TKT2 (knockout), DXPS (overexpressed), PItex (overexpressed), and TPI (underexpressed). The dashed red production envelope is for the design strategy using the maximal regulation set: FUM (knockout), R1PK (knockout), ADK3 (overexpressed), PItex (overexpressed), and ADK1 (underexpressed). Reaction names are consistent with the genome-scale metabolic network model of *E. coli* iML1515.

We have so far assumed that regulation targets can be selected only from the minimal regulation set derived from the first computational step of OptDesign. Under this assumption, it ensures that regulation manipulations are used as few as possible since suggested regulation levels cannot be exactly guaranteed in experimental implementation. However, in the case that multiple metabolic routes exist between two metabolites, the minimal regulation set will have only one of them included. In view of this, we have also computed the maximal regulation set by maximizing the number of reactions that can have noticeable flux changes. Taking lycopene as an example, the number of regulation candidates increases sharply to 119 in the maximal regulation set from 43 in the minimal regulation set (see Supporting Information Data 1). Consequently, the resulting larger solution space makes it possible to identify design strategies with a better minimum guaranteed flux for lycopene (see Figure 4). In both cases, the design strategies identified by OptDesign couple lycopene production with growth, although the strategy from the maximal regulation set yields a higher production rate than that from the minimal regulation set.

**Figure 5:**
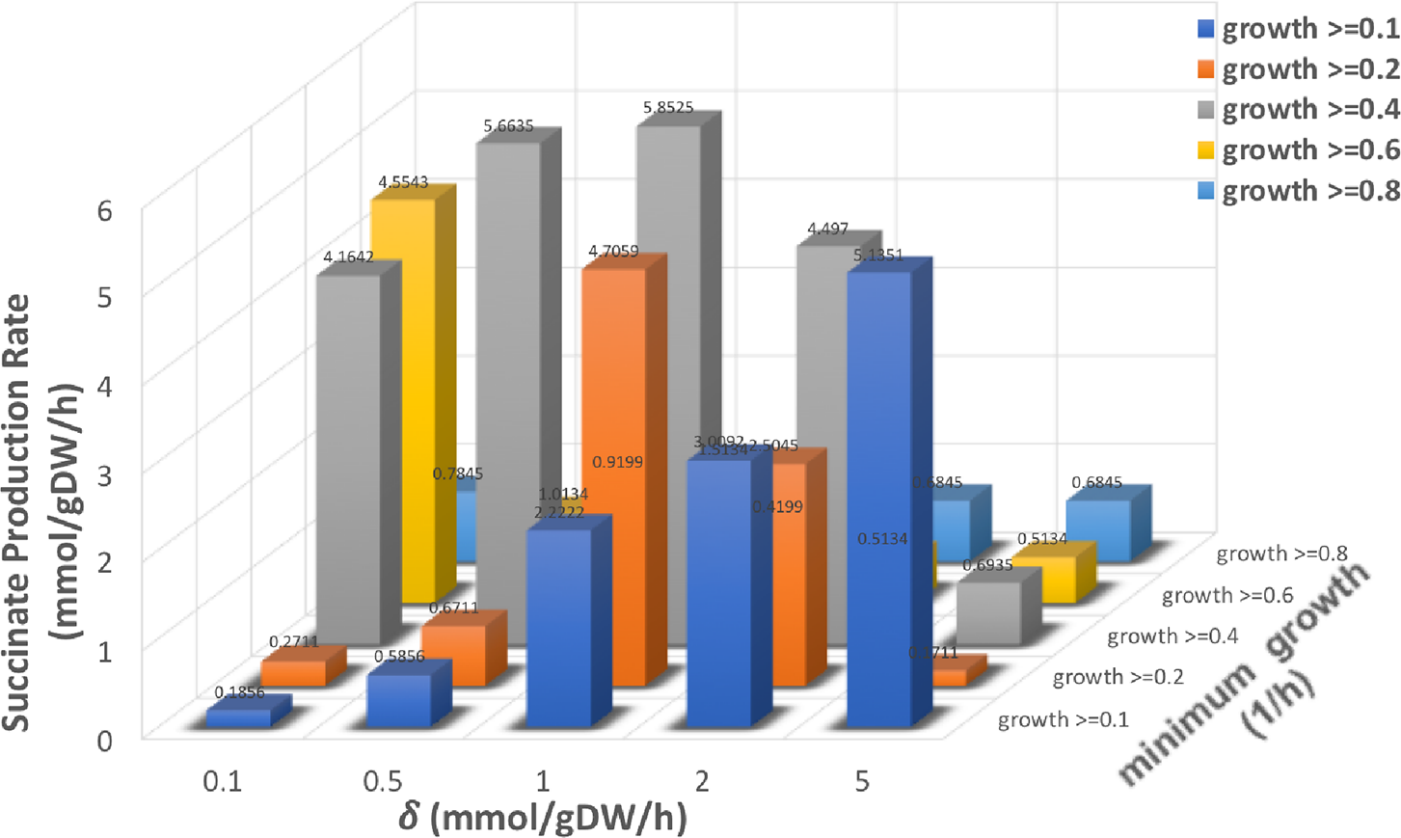
Influence of δ and minimum growth on succinate production.

**Figure 6:**
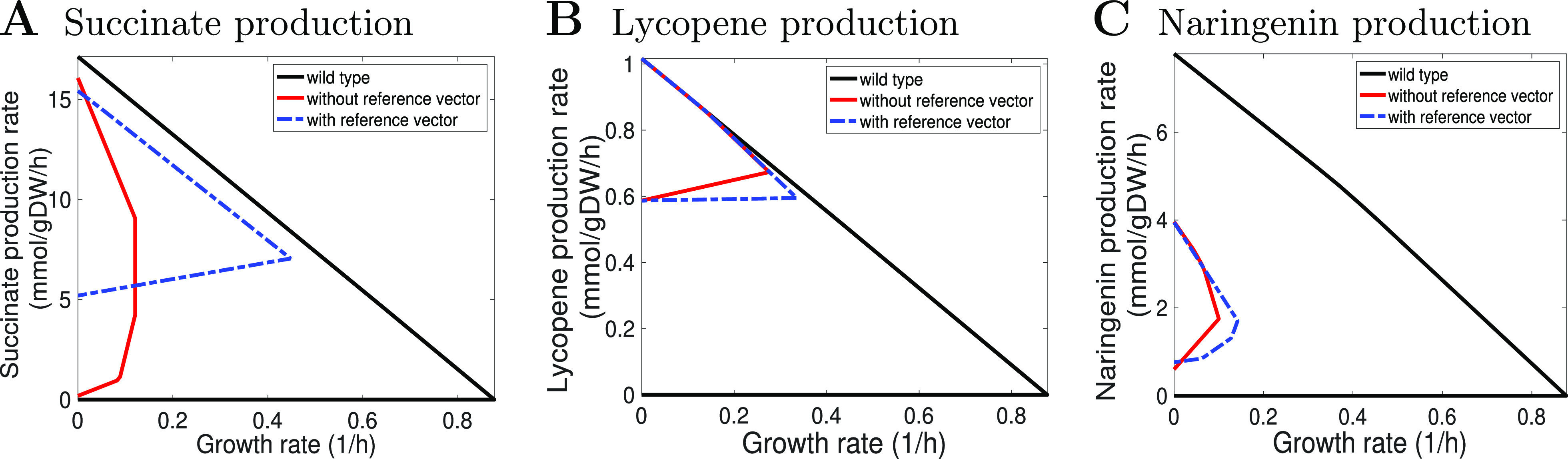
Comparison of production envelopes obtained by OptDesign with and without a reference flux vector for three target products. The reference flux vector for the wild type was computed using parsimonious FBA (pFBA), which minimizes the sum of squared fluxes in the network.^54^

OptDesign has two key parameters, that is, the flux change δ and the minimum required growth rate, which influence the quality of solutions for high production. Figure 5 shows the sensitivity of OptDesign to these two parameters on identifying design strategies for succinate production (sensitivity analysis of these parameters for lycopene and naringenin production can be seen in Supporting Information Data 1). It is observed that high succinate production is achieved near the anti-diagonal line in the 2-D parameter space. Low production strategies are seen when both parameters have either a small or big value. This is because when the minimum required growth is high, there is little room to adjust flux for biochemical production; on the contrary, when the minimum required growth is small, large flux changes (and more regulation candidates to choose as indicated in Supporting Information Data 1) can be made to boost biochemical production. δ impacts on production too as it not only affects the candidate regulation set but also the flux of candidate reactions for regulation on metabolic networks (see details in Supporting Information Data 1). In practice, it requires careful selection of δ and growth threshold to yield optimum design strategies.In addition, OptDesign can be used with a reference flux vector v* easily by binding the flux bounds of the wild type to v* in eq 3. Figure 6 shows the production envelopes of the design strategies, identified with/without the use of in silico reference flux vectors, for three target products. It can be observed that the use of reference flux vectors increases the size of production envelopes, and the maximum growth rate of reference-guided mutants is higher than that of reference-free mutants. This may be explained by the fact that fixing the wild-type flux vector *v* in eq 3 at v* reduces the room for flux adjustments, hence impacting less on growth rate. The production envelopes for succinate also suggest that reference-guided design sometimes could lead to better solutions. Figure 6 also demonstrates the capability of OptDesign to create (strongly) growth-coupled producing strains whose (minimum) target production increases with growth regardless of reference flux vectors.

**Figure 7:**
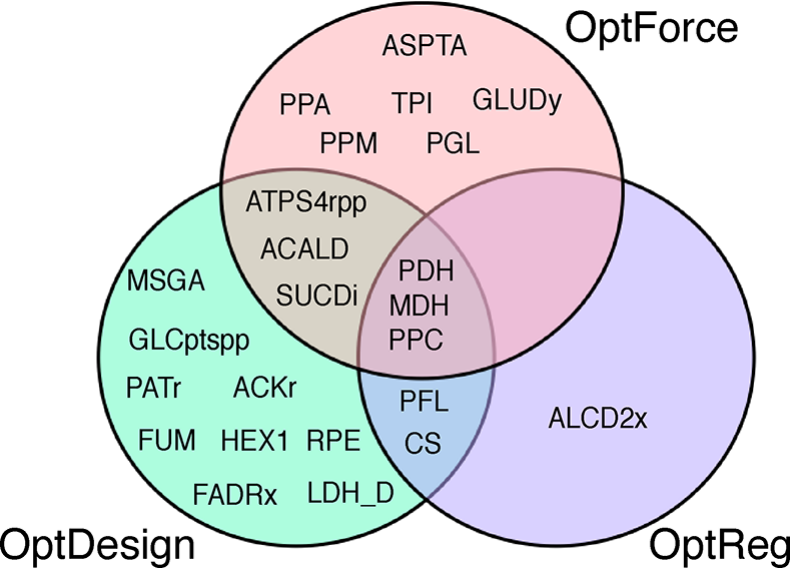
Comparison of different strain design tools without reference flux vectors for succinate overproduction. The intervention targets were identified by using the default genome-scale metabolic network model of *E. coli* iML1515.^32^ A 100% theoretical succinate yield was used in OptForce, and the regulation parameter *C* in OptReg was set to 0.5. Reaction names are consistent with the iML1515 model.

Furthermore, OptDesign highlights the benefit of flux regulation in strain design. For example, with a limit of 5 manipulations (including knockout and flux regulation), OptDesign found numerous design strategies for naringenin, with the best having a minimum guaranteed production flux of 1.73 mmol/g_DW_/h. In contrast, some existing strain design tools (e.g., OptKnock^9^ and NIHBA^22^) using knockout only did not identify any strategies leading to naringenin production. Similar to OptDesign, there also exist a few tools, for example, OptForce^26^ and OptReg,^10^ that can identify both flux regulation and knockout targets. We compared OptDesign with OptForce and OptReg in terms of manipulation targets. For succinate production (see Figure 7), it is noticed that there is a large overlap between the design strategies predicted by these tools, and the common interventions which tend to increase the flux flow toward succinate are all from the core central metabolism, highlighting that intervention of these common targets is effective to increase the availability of succinate precursors. In addition, Figure 7 also shows that OptDesign can find more novel manipulations than the other two tools, demonstrating its capability C1 (Table 1) that enables the search for near-optimal alternatives. OptReg identified fewer regulation targets than the others as it tends to couple the maximum growth rate with target production. OptForce eliminated possible near-optimal but important manipulations by restricting its overproduction target, thereby producing fewer intervention targets than OptDesign.

Finally, OptDesign has been developed to identify metabolic manipulations regardless of whatever the wild-type flux distribution looks like, and it can be used with flux measurements if available. Indeed, a measured wild-type flux vector can help refine the manipulation candidates, leading to a more accurate prediction of design strategies. In addition, the threshold for noticeable flux change defined in this work can be further adjusted with measured data, and different reactions can have distinct values for this parameter. Dedicated threshold values allow for a better prediction of rational flux modifications. Although OptDesign has been implemented for reaction-level phenotype prediction, it can be easily modified to predict design strategies at the gene level. For example, OptDesign can be applied to metabolic network models with an advanced stoichiometric representation of gene–protein–reaction associations,^55^ from which design strategies consisting of gene targets can be identified.

## Supporting Information Available

Bilevel problem reformulation, lycopene and naringenin biosynthetic pathway, model reduction, and impact of OptDesign parameters on biochemical production (PDF)
Comparison between in silico predictions and in vivo manipulations for nine compounds; knockout and regulation candidates for succinate, lycopene, and naringenin; impact of reference flux vectors (XLSX)

## Footnotes

a It is worth noting that the desired down-regulation sometimes may not be achievable when the selective pressure to increase gene expression results in increased fitness.
