# Supplementary material for: OptDesign: Identifying Optimum Design Strategies in Strain Engineering for Biochemical Production
Source: ACS Synth Biol. 2022 Apr 7;11(4):1531–41. doi: 10.1021/acssynbio.1c00610 (PMC9016760; doi:10.1021/acssynbio.1c00610)
Supplement: Supplementary file 1 — sb1c00610_si_001.pdf [file sb1c00610_si_001.pdf]

Supplemental Information

OptDesign: Identifying optimum design strategies in strain engineering for biochemical production

Shouyong Jiang*[1], Irene Otero-Muras[2], Julio R. Banga[3], Yong Wang[4], Marcus Kaiser[5], and Natalio Krasnogor[6]

[1]Department of Computer Science, University of Aberdeen, AB24 3FX, U.K.
[2]Institute for Integrative Systems Biology, UV-CSIC, 46980 Valencia, Spain
[3]Computational Biology Lab, MBG-CSIC, Pontevedra 36143, Spain
[4]School of Automation, Central South University, Changsha 410083, China
[5]School of Medicine, University of Nottingham, Nottingham NG7 2RD, U.K.
[6]School of Computing, Newcastle University, Newcastle upon Tyne NE4 5TG, U.K.

March 18, 2022

This is the supplementary material to the paper entitled "OptDesign: Identifying optimal strain design strategies for biochemical production". This material provides bilevel problem reformulation, lycopene and naringenin biosynthetic pathway, model reduction, and impact of OptDesign parameters on biochemical production

1 MILP Reformulation of Bilevel Optimisation Problem in OptDesign

OptDesign has an important step which is to identify optimal manipulation strategies. This step involves a bilevel problem as follows:

$$\begin{aligned}
\text{BP0:} \quad & \max_{y^+,y^-,y^\times} \min_{v,\Delta v} c_P^T(v+\Delta v) && \text{(1a)}\\
& s.t. \quad \Delta v_j \geq \delta_j y_j^+ + \Delta v_j^{min}(1-y_j^+), j \in F^+ && \text{(1b)}\\
& \Delta v_j \leq -\delta_j y_j^- + \Delta v_j^{max}(1-y_j^-), j \in F^- && \text{(1c)}\\
& lb_j(1-y_j^\times) \leq v_j + \Delta v_j \leq ub_j(1-y_j^\times), j \in F^\times && \text{(1d)}\\
& y_j^+ + y_j^- + y_j^\times \leq 1, j \in J && \text{(1e)}\\
& \sum\nolimits_{j \in F^\times} y_j \leq K_\times && \text{(1f)}\\
& \sum\nolimits_{j \in F^+} y_j^+ + \sum\nolimits_{j \in F^-} y_j^- + \sum\nolimits_{j \in F^\times} y_j^\times \leq K_m && \text{(1g)}\\
& v \in FS_w, v_j + \Delta v_j \in FS_m && \text{(1h)}
\end{aligned}$$

where c_P is a coefficient vector for the target biochemical. $y_j^\times = 1$ represents the knockout of reaction j, leading to zero flux in this reaction as illustrated by constraint (1d). y_j^+ and y_j^- are binary variables representing the flux of reaction j increases and decreases by at least a noticeable level $\delta_j > 0$ from the wild type to the production phenotype, respectively. Equivalently,

*math4neu@gmail.com

$y_j^+ = 1$ (y_j^-=1) implies $\Delta v_j \geq \delta_j$ ($\Delta v_j \leq -\delta_j$). Δv_j^{min} and Δv_j^{max} are the lower and upper bounds of flux change Δv_j, respectively. Special reactions in which fluxes are not allowed to be decreased (increased), e.g., non-growth associated maintenance, should have a zero value for the lower (upper) bound of their corresponding Δv components. Each reaction cannot increase and decrease flux simultaneously, which implies the constraint (1e). Constraints (1f) and (1g) limits the allowable number of knockouts and the total number of manipulations. Constraint (1h) defines the flux space of the wild-type and production strain, respectively. For simplicity, we replace constraint (1h) by $v_{gr} \geq f_{gr}$, $v_p + \Delta v_p \geq f_p$, subject to mass balance and thermodynamic constraints, where f_{gr} and f_p are the growth rate of the wild type and production rate of the mutant, respectively. In other words, $FS_w = \{v \in R^n | Sv = 0, lb_j^w \leq v_j \leq ub_j^w, j \in J\}$, $FS_m = \{v + \Delta v \in R^n | S\Delta v = 0, lb_j^m \leq v + \Delta_v \leq ub_j^m, j \in J\}$, where, $F^+ \cup F^- \cup F^\times$ are the candidate sets of up-regulation, down-regulation, and knockout, respectively. To achieve the smallest set of manipulations, we can modify BP0 slightly by subtracting a new item from the objective as follows:

$$
\begin{aligned}
\text{BP1:} \quad & \max_{y^+, y^-, y^\times} \left(\min_{v, \Delta v} c_P^T(v + \Delta v) - \gamma \sum_{j \in J} (y_j^+ + y_j^- + y_j^\times) \right) && \text{dual variables} && \text{(2a)} \\
s.t. \quad & Sv = 0 && [\lambda^v] && \text{(2b)} \\
& S(\Delta v) = 0 && [\lambda^{\Delta v}] && \text{(2c)} \\
& v + \Delta v \geq lb^m && [\alpha] && \text{(2d)} \\
& v + \Delta v \leq ub^m && [\beta] && \text{(2e)} \\
& v \geq lb^w && [\phi] && \text{(2f)} \\
& v \leq ub^w && [\varphi] && \text{(2g)} \\
& \Delta v_j \geq \delta_j y_j^+ + \Delta v_j^{min}(1 - y_j^+), j \in F^+ && [\zeta] && \text{(2h)} \\
& \Delta v_j \leq -\delta_j y_j^- + \Delta v_j^{max}(1 - y_j^-), j \in F^- && [\eta] && \text{(2i)} \\
& v_j + \Delta v_j = 0, \quad for \ \ j \ \in F^\times \& y_j = 1 && [\pi] && \text{(2j)} \\
& y_j^+ + y_j^- + y_j^\times \leq 1, j \in J && && \text{(2k)} \\
& \sum\nolimits_{j \in F^\times} y_j \leq K_\times && && \text{(2l)} \\
& \sum\nolimits_{j \in F^+} y_j^+ + \sum\nolimits_{j \in F^-} y_j^- + \sum\nolimits_{j \in F^\times} y_j^\times \leq K_m && && \text{(2m)} \\
& && && \text{(2n)}
\end{aligned}
$$

where γ is a significantly small positive value(e.g., 10^{-5}) to favour the search for the solutions with fewer manipulations. Thereafter, we can cast the problem using duality theory [Burgard

et al., 2003] to a single-level mixed-integer optimisation problem (MIP):

$$
\begin{aligned}
\text{MIP:} \quad & \max_{y^+, y^-, y^\times} \; c_P^T(v + \Delta v) - \gamma \sum_{j \in J} (y_j^+ + y_j^- + y_j^\times) \\
& s.t. \quad Sv = 0 \\
& \qquad S(\Delta v) = 0 \\
& \qquad v + \Delta v \geq lb^m \\
& \qquad v + \Delta v \leq ub^m \\
& \qquad v \geq lb^w \\
& \qquad v \leq ub^w \\
& \qquad \Delta v_j \geq \delta_j y_j^+ + \Delta v_j^{min}(1 - y_j^+), j \in F^+ \\
& \qquad \Delta v_j \leq -\delta_j y_j^- + \Delta v_j^{max}(1 - y_j^-), j \in F^- \\
& \qquad v_j + \Delta v_j \geq lb_j(1 - y_j^\times), j \quad \in F^\times \\
& \qquad v_j + \Delta v_j \leq ub_j(1 - y_j^\times), j \quad \in F^\times \\
& \qquad \sum_{i \in M} S_{ij} \lambda_i^v - \alpha_j + \beta_j - \phi_j + \varphi_j = c_{p,j}, j \in J \setminus F^\times \\
& \qquad \sum_{i \in M} S_{ij} \lambda_i^v - \alpha_j + \beta_j - \phi_j + \varphi_j + \pi_j = c_{p,j}, j \in F^\times \\
& \qquad \sum_{i \in M} S_{ij} \lambda_i^{\Delta v} - \alpha_j + \beta_j - \zeta_j + \eta_j = c_{p,j}, j \in J \setminus F^\times \\
& \qquad \sum_{i \in M} S_{ij} \lambda_i^{\Delta v} - \alpha_j + \beta_j - \zeta_j + \eta_j + \pi_j = c_{p,j}, j \in F^\times \\
& \qquad -Hy_j^\times \leq \pi_j \leq Hy_j^\times, j \in F^\times \\
& \qquad -c_P^T(v + \Delta v) = (ub^m)^T\beta - (lb^m)^T\alpha + (ub^w)^T\phi - (lb^m)^T\varphi + (\Delta v^{max})^T\eta \\
& \qquad\qquad - (\Delta v^{min})^T\zeta - (\Delta v^{max} + \delta)^T(y^- \odot \eta) + (\Delta v^{min} - \delta)^T(y^+ \odot \zeta) \\
& \qquad y_j^+ + y_j^- + y_j^\times \leq 1, j \in J \\
& \qquad \sum\nolimits_{j \in F^\times} y_j \leq K_\times \\
& \qquad \sum\nolimits_{j \in F^+} y_j^+ + \sum\nolimits_{j \in F^-} y_j^- + \sum\nolimits_{j \in F^\times} y_j^\times \leq K_m \\
& \qquad y_j^+ \in \{0,1\}, y_j^- \in \{0,1\}, y_j^\times \in \{0,1\}, \lambda_i^v \in \mathbb{R}, \lambda_i^{\Delta v} \in \mathbb{R}, \alpha_j \geq 0 \\
& \qquad \beta_j \geq 0, phi_j \geq 0, \varphi_j \geq 0, zeta_j \geq 0, eta_j \geq 0.
\end{aligned}
$$

where M is the set of indices of metabolites in the network. It can be observed from the above that there are two non-negative nonlinear terms, highlighted in red, in the constraints. Both terms are a product of a binary variable and a continuous variable. i.e., $z = y \cdot w$ (y is binary and $w > 0$ is continuous). We linearise $z_j = y_j w_j$ as follows:

$$z_j \geq 0 \tag{3}$$

$$z_j \leq w_j \tag{4}$$

$$z_j \leq C y_j \tag{5}$$

$$z_j \geq w_j - C(1 - y_j) \tag{6}$$

By defining $z^- = y^- \odot \eta$ and $z^+ = y^+ \odot \zeta$ subject to the above linearisation constraints, the optimisation problem MIP becomes a standard mixed-integer linear program, which can be solved by modern MILP solvers efficiently.

Table S1: Heterologous biosynthesis pathway for naringenin. A list of reactions including exchange ones added to the iML1515 model.

Reaction name (abbrev.)	Reaction formula	Subsystems
EX_cma_e	cma_e$\Longleftrightarrow$	Flavonoid biosynthesis
CMAt	cma_e $\Longleftrightarrow$ cma_c	Flavonoid biosynthesis
CCL	cma_c + atp_c +coa_c $\longrightarrow$ amp_c + cmcoa_c + pi_c	Flavonoid biosynthesis
CHS	3 malcoa_c +cmcoa_c $\longrightarrow$ 4 coa_c + chal_c + 3 co2_c	Flavonoid biosynthesis
CHI	chal_c $\longrightarrow$ narg_c	Flavonoid biosynthesis
NARGt	chal_c $\Longleftrightarrow$ narg_e	Flavonoid biosynthesis
EX_narg_e	narg_e $\Longleftrightarrow$	Flavonoid biosynthesis

Table S2: Heterologous biosynthesis pathway for lycopene. A list of reactions including exchange ones added to the iML1515 model.

Reaction name (abbrev.)	Reaction formula	Subsystems
crtE	frdp_c + ipdp_c $\longrightarrow$ ppi_c + ggdp_c	Carotenoid biosynthesis
crtB	2 ggdp_c $\longrightarrow$ ppi_c + phyto_c	Carotenoid biosynthesis
crtI	8 nadp_c + phyto_c $\longrightarrow$ 8 nadph_c + lyco_c	Carotenoid biosynthesis
LYCOtex	lyco_c $\Longleftrightarrow$ lyco_e	Carotenoid biosynthesis
EX_lyco_e	lyco_e $\Longleftrightarrow$	Carotenoid biosynthesis

2 Biosynthetic Pathways Added to iML1515

Model reduction and knockout candidate selection

Model reduction and candidate selection strategies [Feist *et al.*, 2010; Jiang *et al.*, 2020] are introduced. Specifically, linear reactions (where a metabolite is produced by one reaction and consumed by another) were compressed and only one reaction was selected from a linear reaction group. Dead end reactions which does not carry fluxes were removed from the metabolic network. Reactions which are both computationally and experimentally essential (linked to essential genes) were not considered candidates. Reactions in certain subsystems such as cell envelope biosynthesis, murein biosynthesis are were considered. Exchange reactions and those who does not have any associated genes were also not considered. To further reduce the size of candidates, reactions that participate in high carbon (say 10 carbons) conversion are not likely to carry significant fluxes and therefore were excluded from consideration. As a result, approximately 300 candidates were obtained for the consideration of knockout. Figure S1 illustrates the procedure.

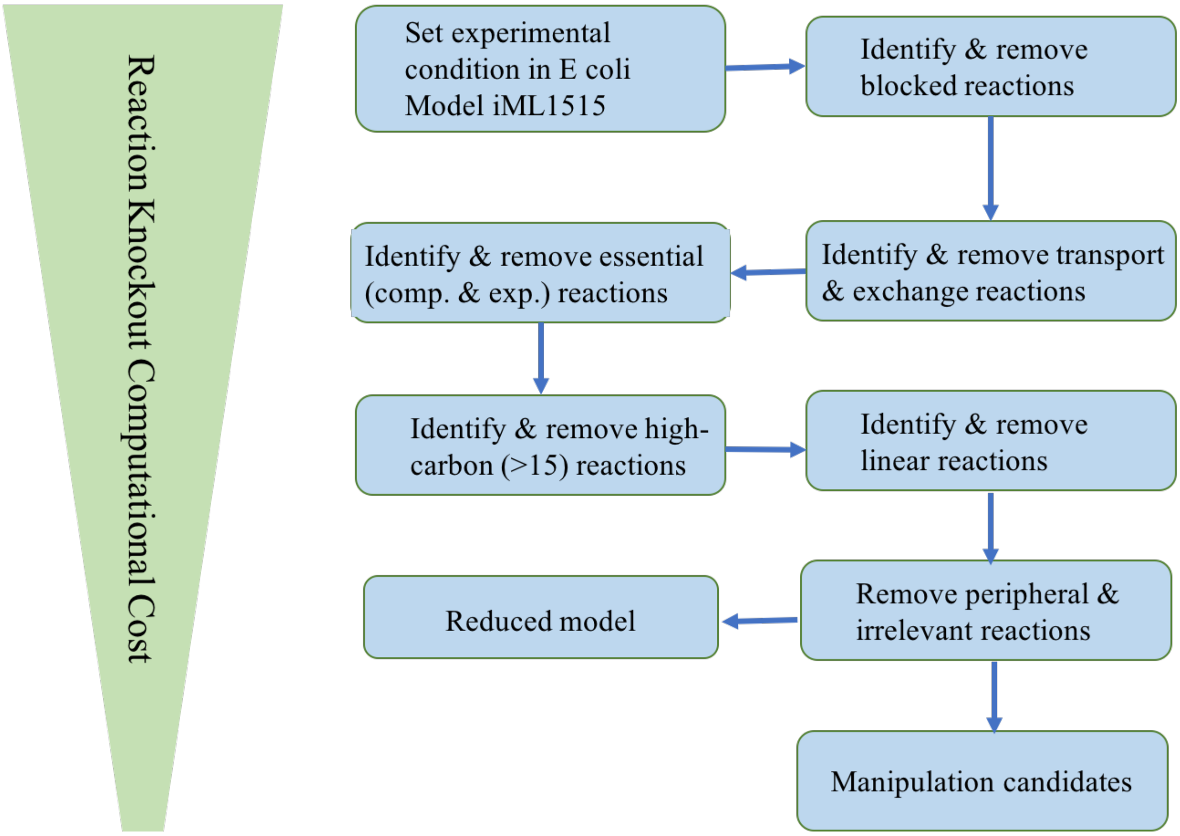

Figure S1: Flowchart of model reduction and candidate selection. The number of knockout candidates (computational cost) reduces with the model reduction steps.

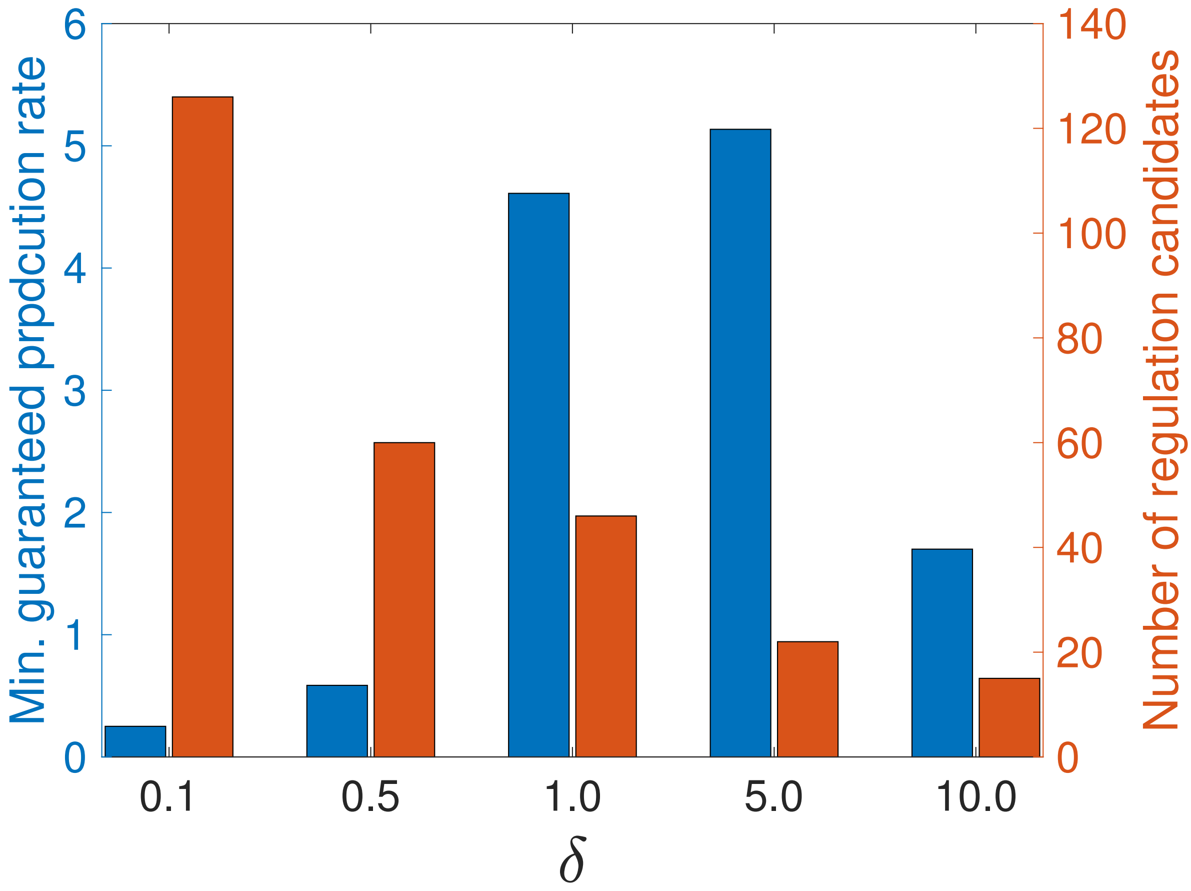

Figure S2: Influence of threshold δ on succcinate production.

References

Anthony P. Burgard, Priti Pharkya, and Costas D. Maranas. Optknock: A bilevel programming framework for identifying gene knockout strategies for microbial strain optimization. *Biotechnology and Bioengineering*, 84(6):647–657, 2003.

Adam M. Feist, Daniel C. Zielinski, Jeffrey D. Orth, Jan Schellenberger, Markus J. Herrgard, and Bernhard Ø. Palsson. Model-driven evaluation of the production potential for growth-coupled products of Escherichia coli. *Metabolic Engineering*, 12(3):173–186, 2010.

Shouyong Jiang, Yong Wang, Marcus Kaiser, and Natalio Krasnogor. NIHBA: a network interdiction approach for metabolic engineering design. *Bioinformatics*, 36(11):3482–3492, 03 2020.

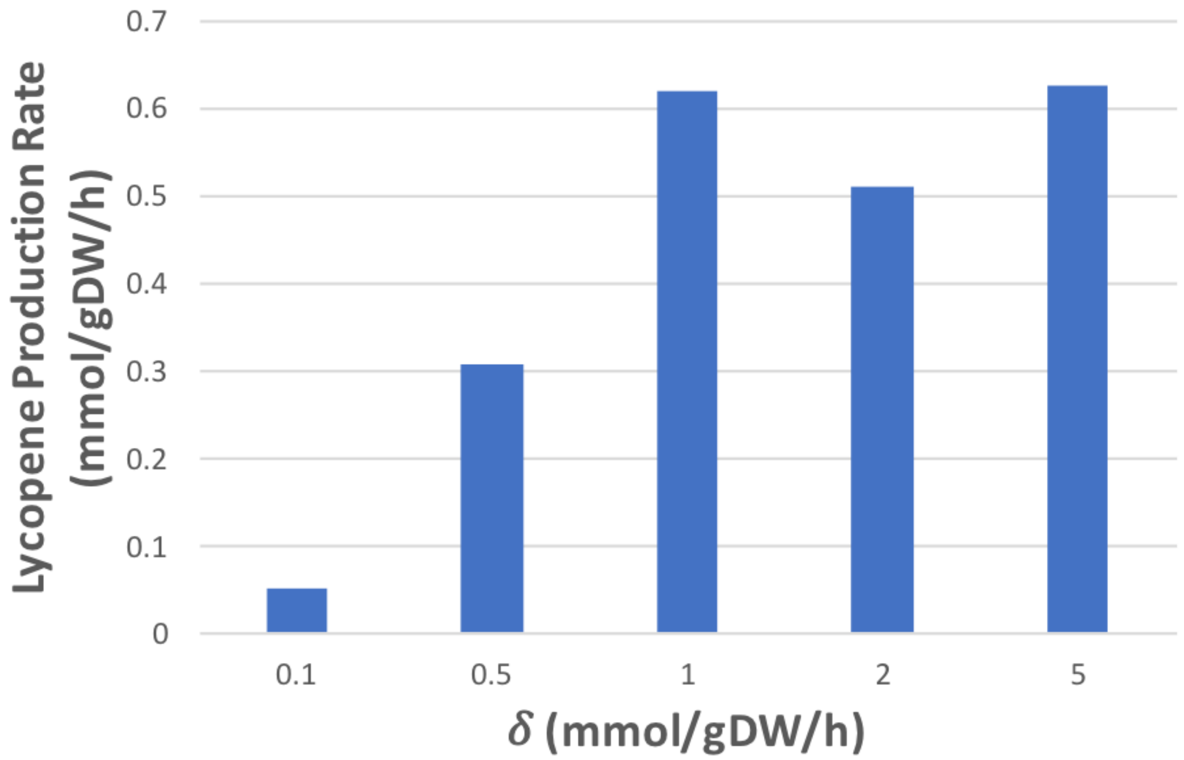

Figure S3: Influence of threshold δ on lycopene production.

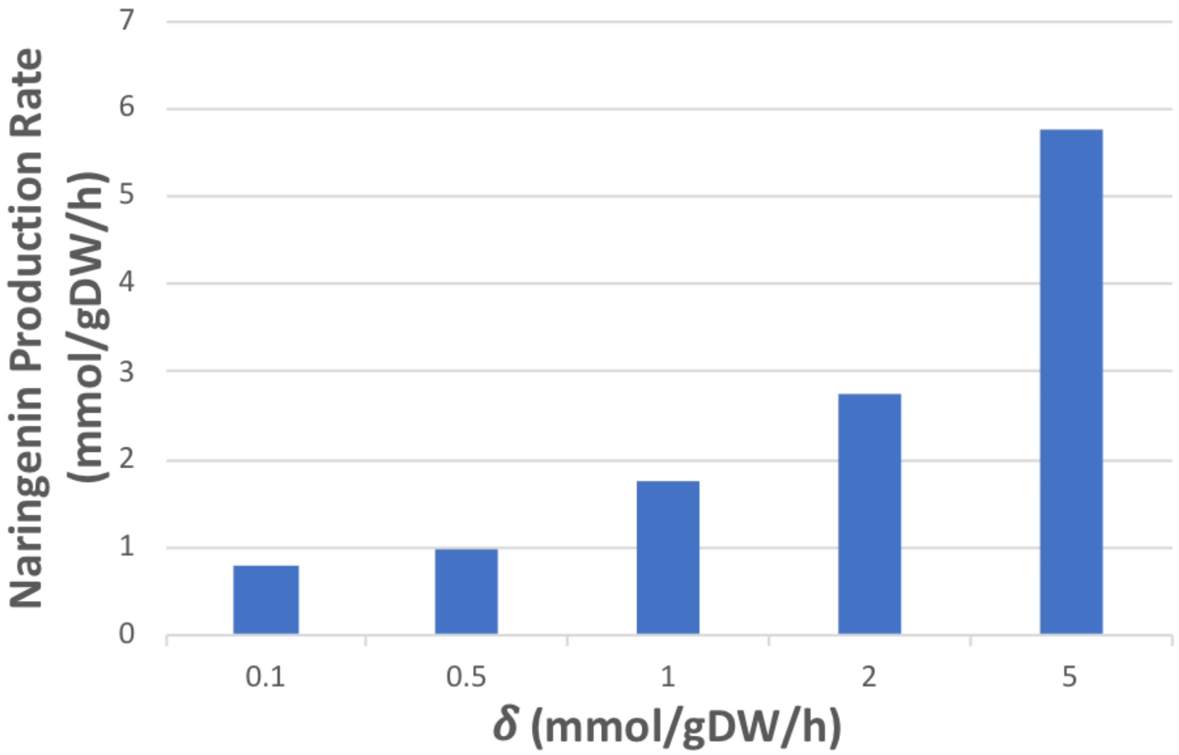

Figure S4: Influence of threshold δ on naringenin production.
